# Imaging and mechanical characterization of different junctions in spider orb webs

**DOI:** 10.1038/s41598-019-42070-8

**Published:** 2019-04-08

**Authors:** Gabriele Greco, Maria F. Pantano, Barbara Mazzolai, Nicola M. Pugno

**Affiliations:** 1 Laboratory of Bio-Inspired & Graphene Nanomechanics, Department of Civil, Environmental and Mechanical Engineering, University of Trento, Via Mesiano, 77, 38123 Trento, Italy; 2 Center for Micro-BioRobotics@SSSA, Istituto Italiano di Tecnologia, Viale Rinaldo Piaggio 34, I-56025 Pontedera, Italy; 3 School of Engineering and Materials Science, Queen Mary University of London, Mile End Road, E1 4NS London, United Kingdom; 4 Ket-Lab, Edoardo Amaldi Foundation, Via del Politecnico snc, 00133 Rome, Italy

## Abstract

Spider silk and spider orb webs are among the most studied biological materials and structures owing to their outstanding mechanical properties. A key feature that contributes significantly to the robustness and capability to absorb high kinetic energy of spider webs is the presence of junctions connecting different silk threads. Surprisingly, in spite of their fundamental function, the mechanics of spider web junctions have never been reported. Herein, through mechanical characterization and imaging, we show for the first time that spider orb webs host two different types of junction, produced by different silk glands, which have different morphology, and load bearing capability. These differences can be explained in view of the different roles they play in the web, i.e. allowing for a localized damage control or anchoring the whole structure to the surrounding environment.

## Introduction

**Figure 1:**
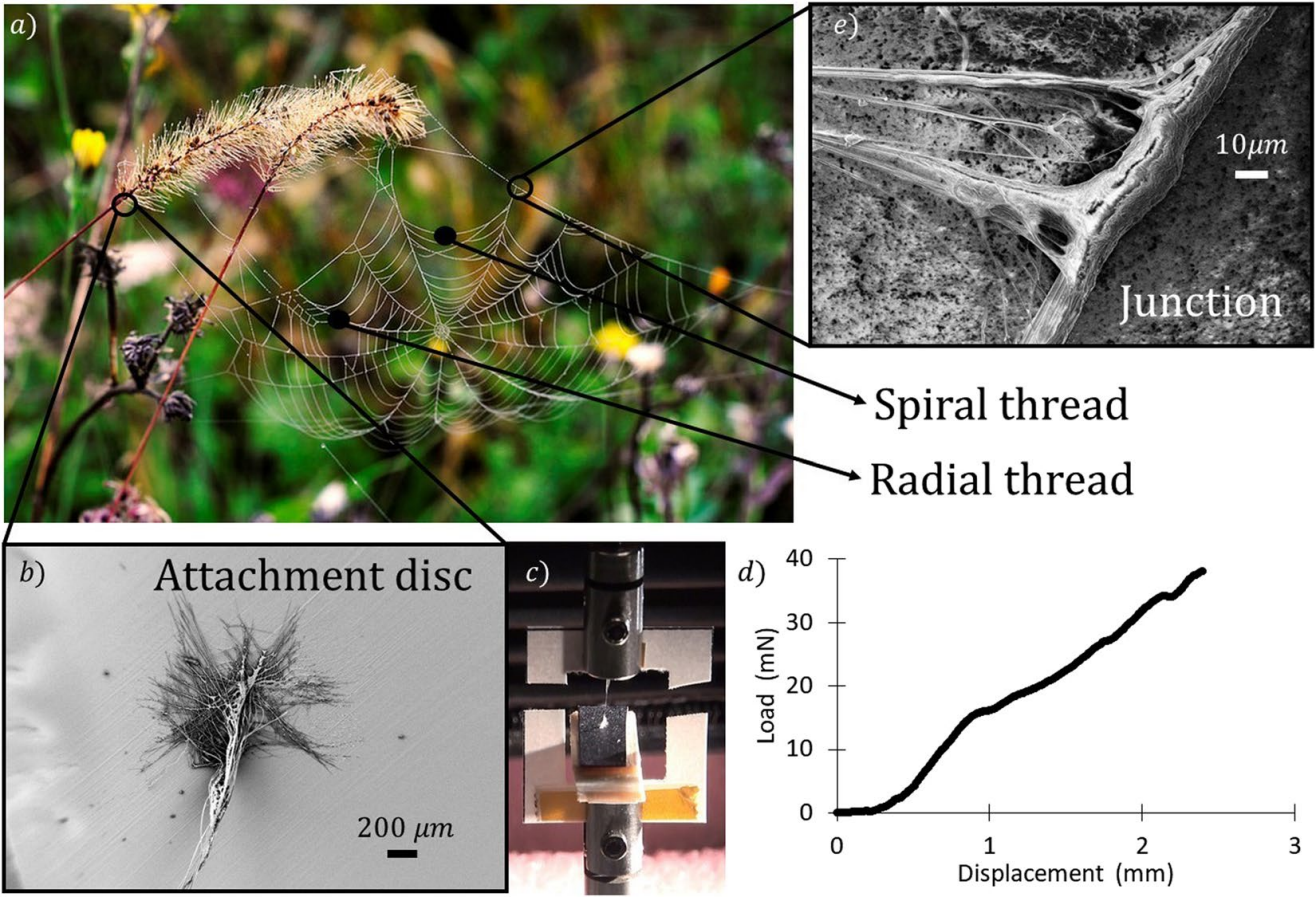
(**a**) Picture of an orb-web structure (courtesy of Federica Fabietti), including radial and spiral threads, as indicated by the arrows. Adjacent threads are held together at junction points. The whole web structure is fixed to the ground by attachment discs. (**b**) SEM picture of an attachment disc, produced by Nuctenea umbratica that anchors the web to a substrate. (**c**) A web anchorage under mechanical characterization through a nanotensile testing machine. Here a dangling silk fiber connected to the disc is pulled until the disc is completely detached from its paper substrate, providing the load-displacement curve reported in (**d**). (**e**) SEM picture of a junction connecting a spiral and a radial thread isolated from an orb web produced by the species Nuctenea umbratica; the junction was not metalized.

Spider silk is a unique protein-based material produced by the silk glands of spiders^1–3^. Owing to its outstanding mechanical properties, which have been investigated in depth^4–8^, spider silk is currently under consideration for different applications, ranging from engineering to medicine^9–13^. During their evolution, spiders have used their silk for a variety of purposes, including production of egg sacs, mating behavior, self-defense and prey capture^1,2,14^. For the capture of prey, the orb-web (Fig. 1a) is one of the most effective structures developed by nature^15^.

From a mechanical point of view, the behavior of the orb-web under external loads due to wind and impacts has been studied mainly through a numerical approach^16,17^. It was found that the spider’s web can resist extreme wind conditions owing to the presence of specific anchorages that securely fix it to a surface^18^. From a structural point of view, these anchorages, or attachment discs, consist of very thin (~tens of nanometers in diameter) fibers embedded in a matrix with an unknown chemical composition^19–21^ (Fig. 1b). The mechanical properties of such discs were intensively investigated through both theoretical and experimental studies. In particular, their mechanical behavior was studied through the theory of multiple peeling and its numerical implementation^22,23^. More recently, experiments were performed in order to derive the peeling force required to detach spider silk anchorages from different substrates (Fig. 1b–d)^24–26^.

Besides anchorages, other important features contribute significantly to the performances of the spider’s web. These are the junctions connecting different threads to one another (Fig. 1e)^27^ within the same web (Fig. 1a). At junctions, web threads interact in a synergistic fashion that provides the whole structure with its unique capability of minimizing the area damaged by the impact of an object, e.g. flies^17^. However, despite their fundamental role, spider web junctions have never been deeply characterized. Thus, herein we focus specifically on the role played by these structural elements and we give special attention to their morphology and mechanical properties.

**Figure 2:**
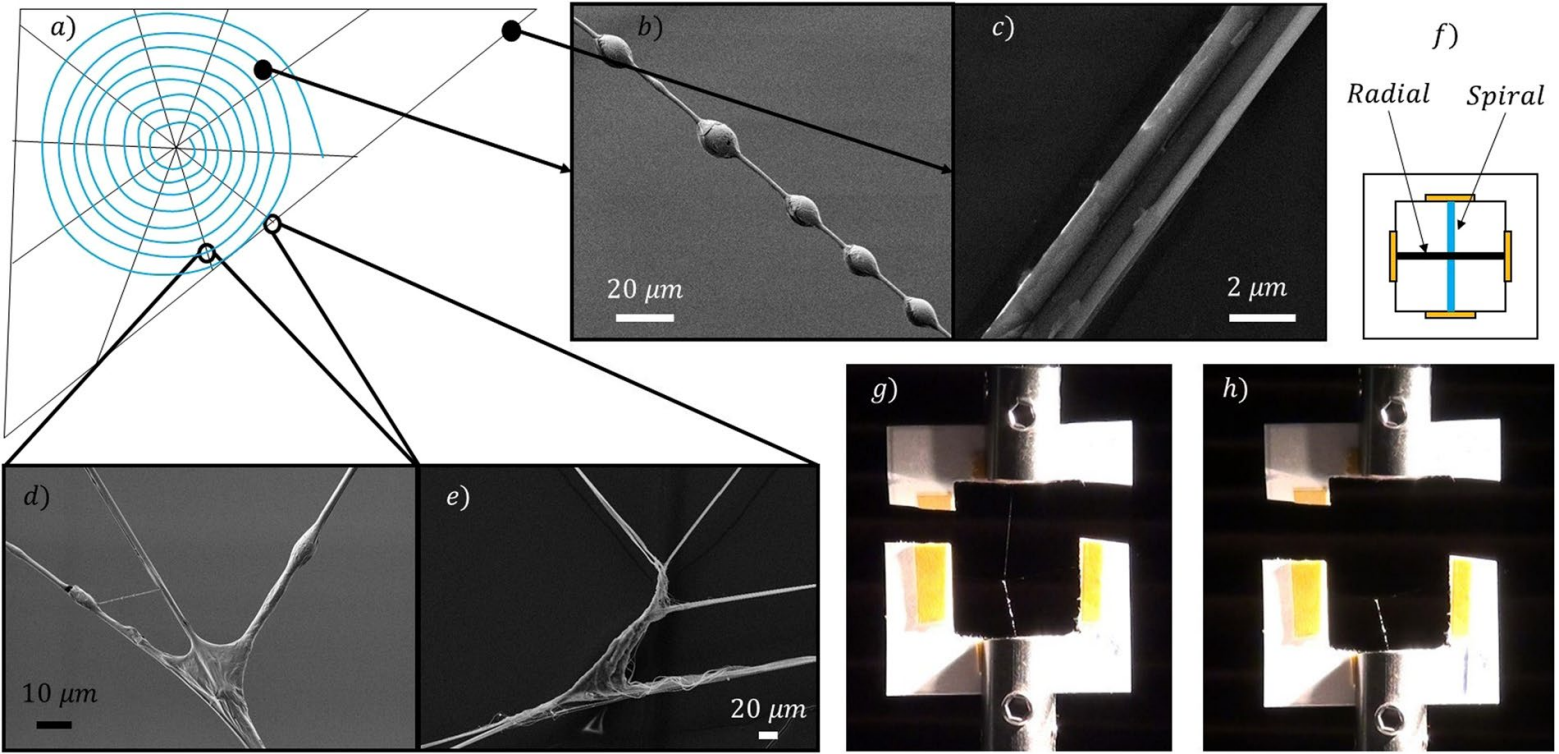
(**a**) Schematic of an orb web, consisting of spiral (**b**) and radial (**c**) threads. (**d**) SEM picture of a junction involving two spiral threads and one radial thread. (**e**) SEM picture of a junction between two radial threads. Significant difference can be observed between the two types of junction. (**f**) Schematic of a web sample mounted on a paper frame to test junction structural properties. (**g**) Junction sample under mechanical characterization through a nanotensile testing machine and (**h**) the remaining structure after junction failure.

In the literature, it is commonly accepted that junctions result from localized deposition of a silk-secretion produced by the piriform glands of spiders^2^, which are the same glands that produce the silk used for the attachment discs (Fig. 1b,e). However, the experimental evidence of this work, in agreement with Vasanthavada *et al*.^28^, shows that aggregate glands, which are normally used to produce the glue droplets in the catching spiral threads^29^ (Fig. 2a,b), are involved, too.

Finally, in order to explain the need for the presence of two different junctions in the orb web, we performed, for the first time, both morphological analyses and mechanical characterization tests.

## Results

**Figure 3:**
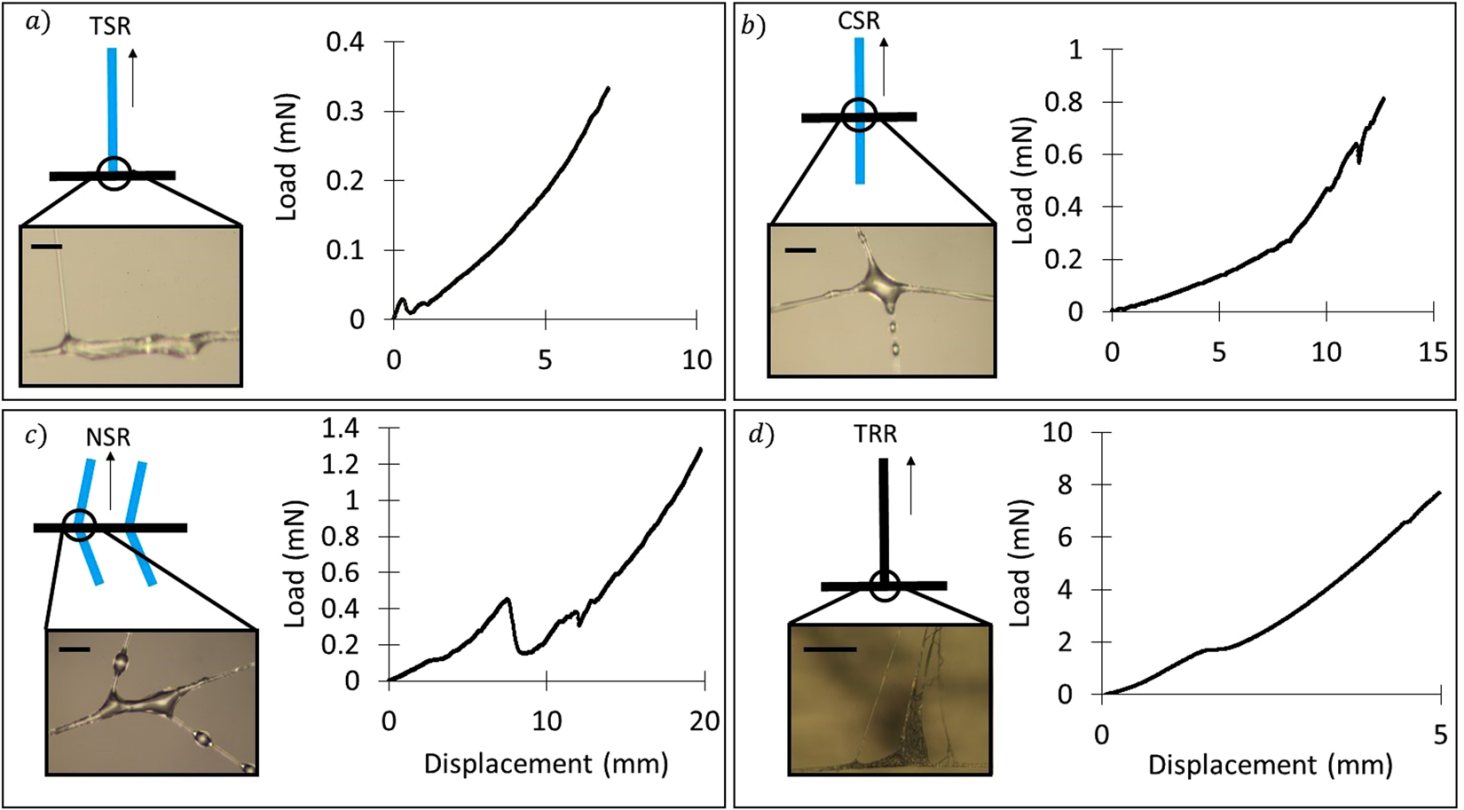
Example Load/Displacement curves derived from testing different web samples, each involving a different number/type of silk threads and junctions. These curves were obtained by pulling until complete detachment: (**a**) a spiral thread from a radial thread; (**b**) a spiral thread from a radial thread connected to another spiral thread; (**c**) a spiral thread form a complex system with more than one spiral thread connected to a single radial thread; (**d**) a radial thread from a radial thread. Images reveal that junctions involving either spiral and radial threads or only radial threads are characterized by different morphology. Scale bar: 10 μm.

In the web structure we can identify two kinds of junction according to the type of the involved threads (either spiral or radial, Fig. 2b,c). If we compare their morphology (Fig. 2d,e and 3), several differences emerge. Junctions between radial threads, which are those occurring along the edges of the web (Fig. 2e) or where this is fixed to the substrate, have a multiple fibril shape. This morphology is similar to that of the attachment disc (Fig. 1b) and could be thus produced by the piriform glands as well^2^. On the other hand, junctions that involve both radial and spiral threads look completely different (Fig. 2d). Indeed, they consist of a glue drop that looks like the droplets in catching spiral threads (Figs 2b and 3a–c). Thus, similarly to these latter, they could be produced by aggregate glands, too. The role of the aggregate glands in the production of this kind of junctions in orb web has never been reported, but only observed in 3D cob web by Vasanthavada *et al*.^28^.

In order to evaluate the mechanical properties of all the orb web junctions, we isolated from the orb web five different samples, each involving a specific number of junctions and silk threads (Fig. 3): (a) T-shaped spiral-to-radial junction (TSR), (b) Cross-shaped spiral-radial junction (CSR), (c) Net-shaped spiral-to-radial junction (NSR), (d) T-shaped radial-to-radial junction (TRR), and radial-from-surface junction (RFS, here reported for the sake of comparison with the literature). The first tested configuration (a) consists of a spiral thread joined nearly perpendicular to a radial thread by aggregate silk glue (Fig. 3a). The second (b) is similar to the previous one, but with another spiral thread that is arranged normal to the radial thread, thus resulting in a cross shape with aggregate glue at the middle (Fig. 3b). The third tested configuration (c) belongs to a more complex web structure, composed of a supporting radial thread that crosses four perpendicular spiral threads that are connected through aggregate silk (Fig. 3c). The fourth tested configuration (d) has a T shape that differs from type (a), as it consists of only radial threads joined together by a multifibril structure that recalls the piriform silk morphology (Fig. 3d). The fifth sample (Fig. 1b–d) is the whole attachment disc of the web as produced by the piriform gland.

**Figure 4:**
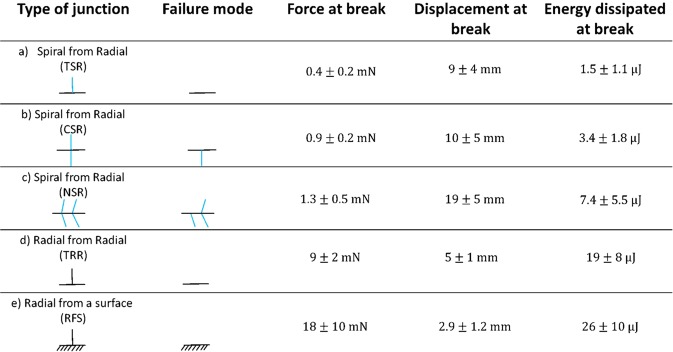
Maximum load/displacement and toughness obtained after testing different web samples. All displacements are referred to an initial length of 0.5 cm. The second column reports what is left after the sample failure: in all the tested configurations, the supporting structure resulted unbroken after the junction failure (i.e., complete detachment of the pulled thread). The toughness was computed by calculating the area under the load-displacement curves.

In order to investigate the mechanical properties, ten replications for each junction typology were considered, where either a spiral or radial thread was pulled out of its junction (Figs 2f–h, S1–S6, Tables S1–S5 and Videos S1–S5). We found that the mean force necessary to break TSR junctions (type a) was 0.4 ± 0.2 mN, with dissipated energy of 1.5 ± 1.1 μJ and mean displacement at break of 9 ± 4 mm (Fig. 4). We noticed that after failure the supporting radial thread remained undamaged (Fig. S2). For the CSR junction (type b), we found that the mean force at break was 0.9 ± 0.2 mN, its dissipated energy was 3.4 ± 1.8 μJ and its mean displacement at break was 10 ± 5 mm (Fig. 4). The supporting structure resulted unbroken after the break of the junction also in this case (Fig. S3). The NSR sample (type c) showed an average force at break of 1.3 ± 0.5 mN, dissipated energy of 7.4 ± 5.5 μJ with the corresponding mean displacement at break being 19 ± 5 mm (Fig. 4). After the rupture of the junction linked to the spiral thread where the force was applied, the remaining structure resulted undamaged (Fig. S4). The mean maximum load that the TRR junction (type d) can withstand was 9 ± 2 mN, its dissipated energy was 19 ± 8 μJ with a corresponding displacement at break of 5 ± 1 mm (Fig. 4). The supporting radial thread resulted undamaged after the junction failure (Fig. S5). Finally, the anchorage sample showed an average force at break of 18 ± 10 mN, dissipated energy of 26 ± 10 μJ, with the corresponding mean displacement at break being 2.9 ± 1.2 mm (Fig. 4). The initial length of the pulled thread was 0.5 cm in all the tested specimens.

In order to interpret the differences among the force values recorded for each junction sample, we performed both ANOVA and Weibull Statistics analysis (Tables S1–S5, S9 and Supporting Figs S6–S11). In particular, junction separation forces differed significantly across all junction types (ANOVA, p ~ 0, all post-hoc pairwise comparisons p < 0.05). Interestingly, the highest p-value (~0.02) was observed for CSR and NSR junctions (Table S9). Indeed, the Weibull distributions obtained from the data available for CSR and NSR samples are very similar, while the distributions corresponding to the samples that involve radial threads show a much larger difference with respect to those that involve spiral threads.

## Discussion

Two features common to all the tested samples are concerned with the maximum force, which is recorded when the pulled thread detaches completely from its junction (Figs S2–S5 and Videos S1–S5), and the shape of the sample after the mechanical test, whose structure (consisting of either another thread or a more complex architecture) looks the same as before the test, except for the pulled thread. This indicates the ability of the web structure to localize the damage regardless the size of its analyzed portion.

Regarding the structural properties collected in Figs 3 and 4, we can immediately see that junctions involving only radial threads, such as TRR or RFS configurations, which are mainly composed of piriform silk, are much stronger (23 times and 46, respectively, compared to TSR) yet tougher (with a dissipated energy of 13 and 26 times bigger than that of TSR sample, respectively) than those involving spiral threads, which are mainly composed of aggregate silk. Such result is also supported by the statistical analysis of the breaking force (Tables S1–S5, S8 and Figs S6–S11). This can be easily explained by recalling the different position that such junctions occupy within the web structure. In particular, junctions between radial threads, which are located at the edge of the web, must support higher loads (e.g. high-speed wind) in comparison to the region embedded within the boundary of the web that has mainly the function to stop preys. In this region, aggregate silk joins the spiral threads to radial threads, providing the spider’s web with its classical orb shape. Spiral-radial thread junctions act as filters for choosing the appropriate prey for the spider and for avoiding useless damage of the cobweb. As big loads could be fatal to the structure, causing an extended and irreversible damage, high kinetic energy objects are allowed to pass through the web by producing a localized rupture, e.g. of a single junction (that thus behaves as a sacrificial element). The existence of two different types of junction becomes thus necessary, with one (i.e., pyriform based junction) for sustaining high loads and holding the structure as a whole and the other one (i.e., aggregate based junction) for catching preys without compromising the integrity of the entire structure and allowing spiders to save energy in rebuilding the web after an impact^17^. Finally, it can be observed from our tests that when we pull a radial thread out of a spiral-radial thread junction, the stiffness of the supporting structure plays a role in determining the mechanical properties of the web sample. Indeed, moving from TSR to CSR and NSR, the increase of structural complexity and stiffness, given by the addition of one or more spiral threads connected to the same radial thread, leads to an increase of the maximum force, and also an increase of displacement at break and dissipated energy (Tables S6–S8). This agrees also to what found for configuration TRR, that is stiffer and tougher than TSR sample. Radial threads are themselves stiffer than spiral ones, with a Young modulus of ~1 GPa, that is one order of magnitude bigger than that of spiral threads (~0.1 GPa). By referring to the force increase in CSR (2.4) and NSR (3.3) with respect to TSR samples (Table S5), this tends to saturate with the increase of complexity (e.g., stiffness) of the structure itself (the force at break of NSR is only 1.4 bigger than that for CSR in spite of the addition of a number of threads). This suggests that after a certain number of threads added to the anchor, there is no more increase in the junction load bearing capacity.

## Conclusion

A 400 million years evolution has designed the orb web as a structure simultaneously able to stop flying prey, localize damage after impacts and withstand high loads. The interaction among the threads and their anchorages to the substrate could provide an explanation to the mechanical efficiency of the orb webs. This interaction is mediated by the presence of junctions that connect threads to each other or to the surrounding environment. In this work, we observed that two different types of junction exist and we measured the force necessary to break them in different configurations. The first type seems to be made of aggregate silk that is used by spiders for joining radial and spiral threads as well as to provide spiral threads with sticky droplets. The second type seems to be composed of pyriform silk that is mainly used for joining either radial threads together and/or radial threads with the substrate. The first type of junction showed a lower breaking force with respect to the second type, even if an increase in both the force and displacement at break was observed as associated to an increased structural complexity. This difference in terms of breaking force can be explained by considering the different roles that these kinds of junction play in the orb web. Indeed, junctions between two radial threads and between radial threads and surfaces have to withstand higher loads since these support the whole web structure, while junctions involving spiral threads have to guarantee damage localization (e.g., web robustness), thus breaking at lower loads.

Our work could provide new information that shed light on the mechanical behavior of spider’s orb web and could be used for the design of new bio-inspired nets and fabrics with superior mechanical properties.

## Methods

### Spiders care and web production

The spider under study was *Nuctenea umbratica* (Clerck 1876), common nocturnal spider that usually builds its web during the night. We kept three individuals in glass terrarium of about 30 × 30 × 40 cm. All spiders were adult females and fed with a weekly diet of *Blaptica dubia* that were breed in the “Laboratory of Bio-inspired and Graphene Nanomechanics” and fed weekly with carrots and fish food. All terrariums were set in the same way with three long sticks covered with paper attached to the wall of the terrarium. Each terrarium was provided with a small refuge, made of paper, in the right corner of the cage to allow the spider to feel protect and live without stress during the day. Each spider produced its orb web after a few days.

### Sample preparation

The tested samples were prepared by following the same procedure reported by Blackledge *et al*.^30^ and Grawe *et al*.^24^ We stuck the web samples on a paper frame provided with a square window of 1 cm side. The web sample was fixed to the paper frame with a double-sided tape. We checked the direction of the spinning process before collecting web samples. By referring to the anchorages, we stuck black paper on the terrarium walls where the spider spins the attachment disc. Then we cut the portion of the paper containing the anchorage and fix it on a wood block (2 × 0.5 × 0.5 cm^3^). This block was fixed to the previous paper support by attaching the radial thread to the upper part of the frame.

### Optical and SEM images

For the morphology characterization, we used an Optical microscope (Jenavert) with a 20x enlargement lens. The microscope was provided with a camera (Canon) connected to a computer for remote control.

For the SEM characterization, we used a Zeiss – 40 Supra. The metallization was made by using a sputtering machine Q150T and the sputtering mode was Pt/Pd 80:20 for 5 minutes. For the Fig. 1b we did not metalize the sample and the picture was taken at 2.0 kV, 1300 Magnification with secondary electron detector. For the Fig. 1c the picture was taken at 2.0 kV, 103 Magnification and with secondary electron detector. With reference to Fig. 2b–e the pictures were taken at 2.0 kV, 15 kV, 2.0 kV and 15.0 kV, 1290, 5580, 2520 and 641 magnifications, respectively, with the secondary electron detector in all cases.

### Mechanical characterization

For the mechanical characterization, we used a nanotensile testing machine (Agilent technologies T150 UTM) with a load cell of 500 mN. The displacement speed was 10 μm per second with the frequency load at 20 Hz. The samples were mounted in order to pull a spiral or radial thread out (depending on the sample) in the direction opposite to spider spinning. The declared sensitivity of the machine is 10 nN for the load and $1\,\dot{{\rm{A}}}$ for the displacement in the dynamic configuration. The tests were recorded with a Sony Camera. Ten samples were tested for each configuration.

## Supplementary information

Supplementary video 1

Supplementary video 2

Supplementary video 3

Supplementary video 4

Supplementary video 5

Supplementary information

## Data Availability

The authors declare that the data supporting the findings of this study are available within the article and its supplementary information files.
