# Supplementary material for: Imaging and mechanical characterization of different junctions in spider orb webs
Source: Sci Rep. 2019 Apr 8;9:5776. doi: 10.1038/s41598-019-42070-8 (PMC6453893; doi:10.1038/s41598-019-42070-8)
Supplement: Supplementary file 6 — Supplementary information [file 41598_2019_42070_MOESM6_ESM.pdf]

Supplementary information

Imaging and mechanical characterization of different junctions in spider orb webs

Gabriele Greco [1, 2]; Maria F. Pantano [1]; Barbara Mazzolai[2] and Nicola Pugno [1, 3, 4*]

[1] Laboratory of Bio-Inspired & Graphene Nanomechanics, Department of Civil, Environmental and Mechanical Engineering, University of Trento, Via Mesiano, 77, 38123 Trento, Italy

[2] Center for Micro-BioRobotics@SSSA, Istituto Italiano di Tecnologia, Viale Rinaldo Piaggio 34, I-56025 Pontedera, Italy

[3]School of Engineering and Materials Science, Queen Mary University of London, Mile End Road, E1 4NS London, United Kingdom

[4]Ket-Lab, Edoardo Amaldi Foundation, Via del Politecnico snc, 00133 Rome, Italy

*Corresponding author: nicola.pugno@unitn.it

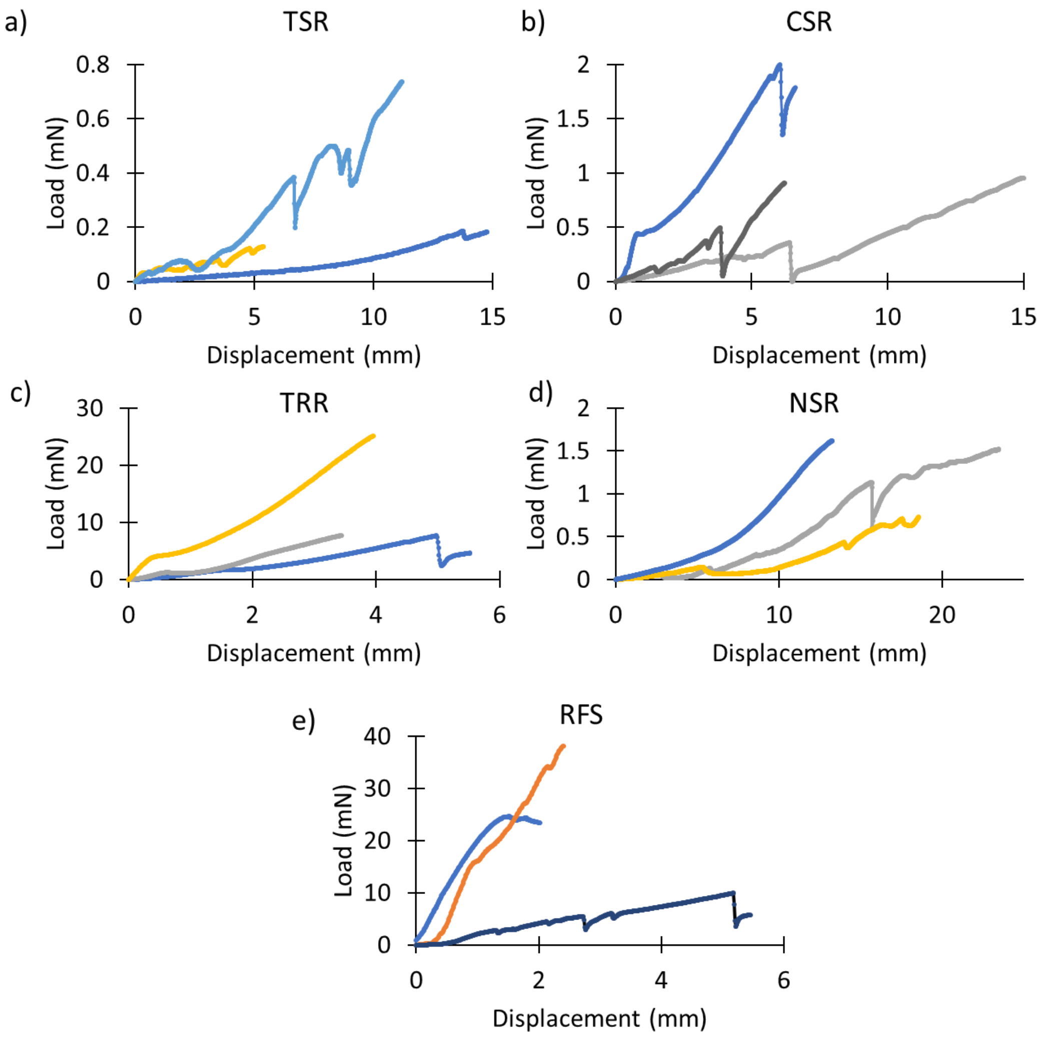

Figure S1: Three representative load-displacement curves observed for a) TSR, b) CSR, c) TRR, d) NSR, e) RFS samples.

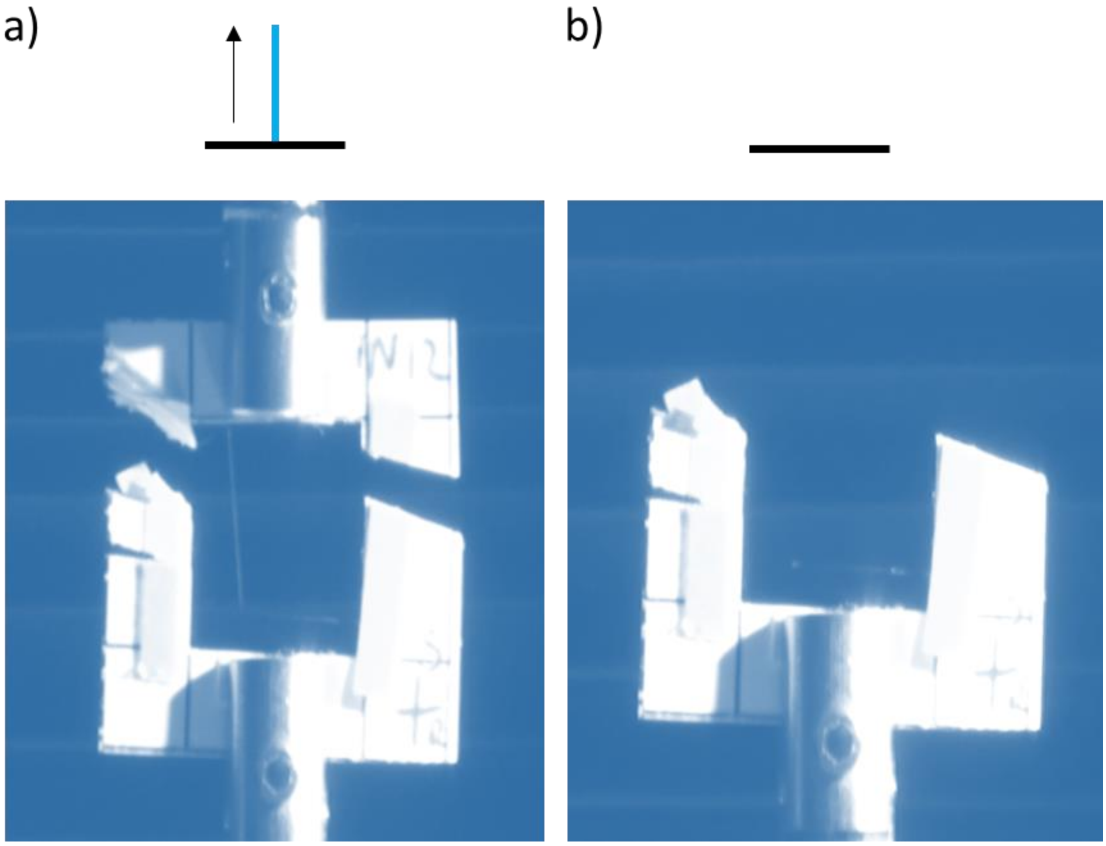

Figure S2: A TSR sample before (a) and after the test (b).

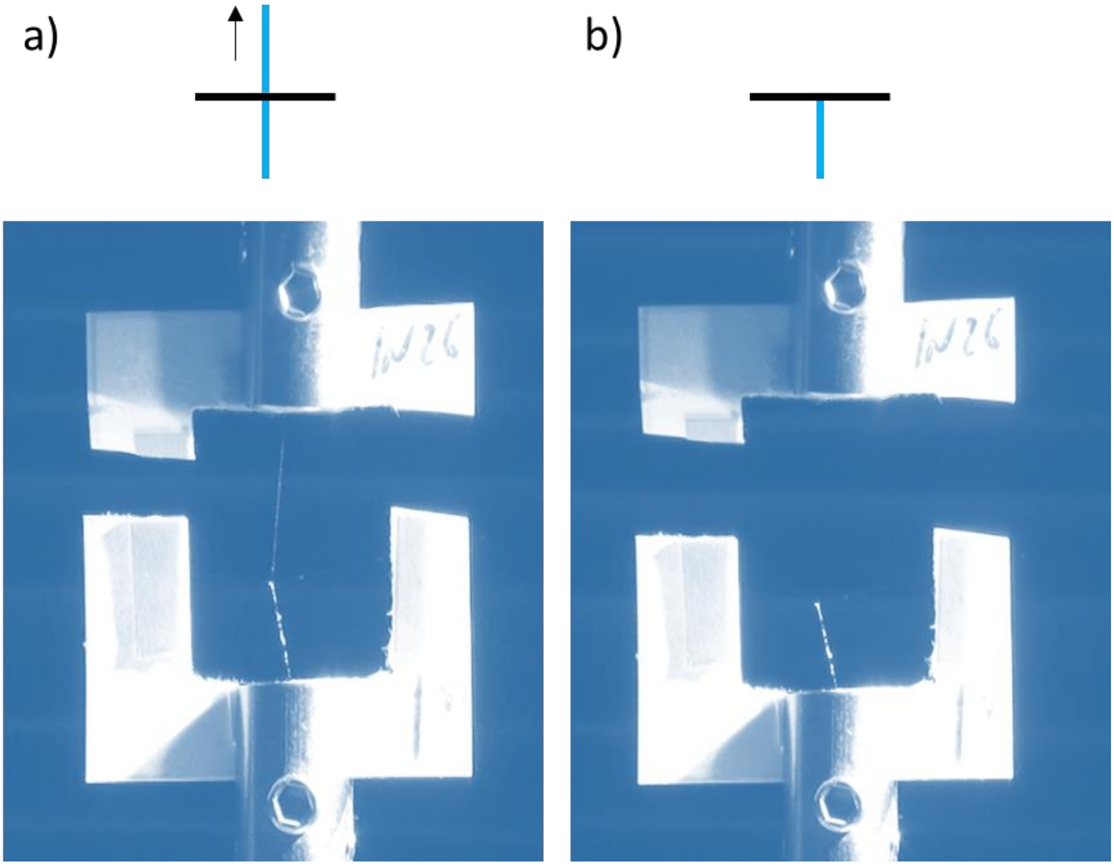

Figure S3: A CSR sample before (a) and after (b) the test.

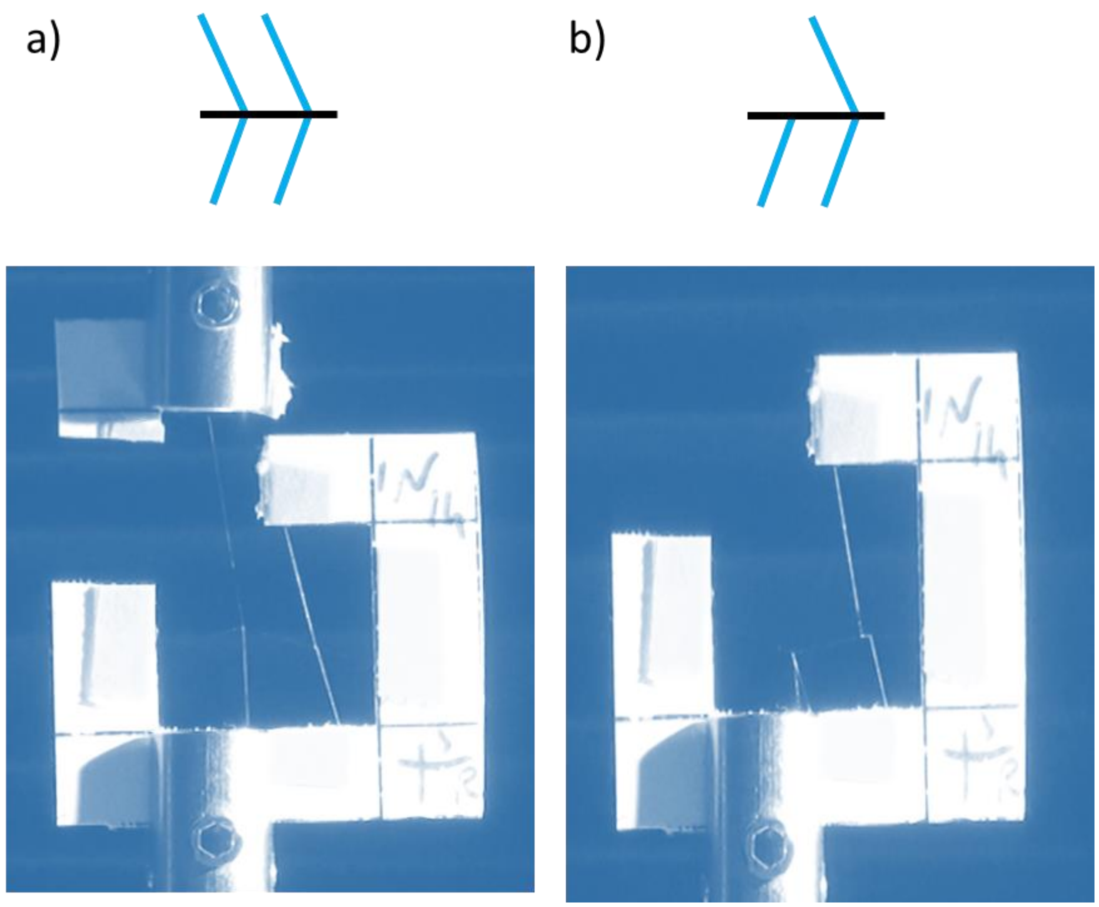

Figure S4: An NSR sample before (a) and after (b) the test.

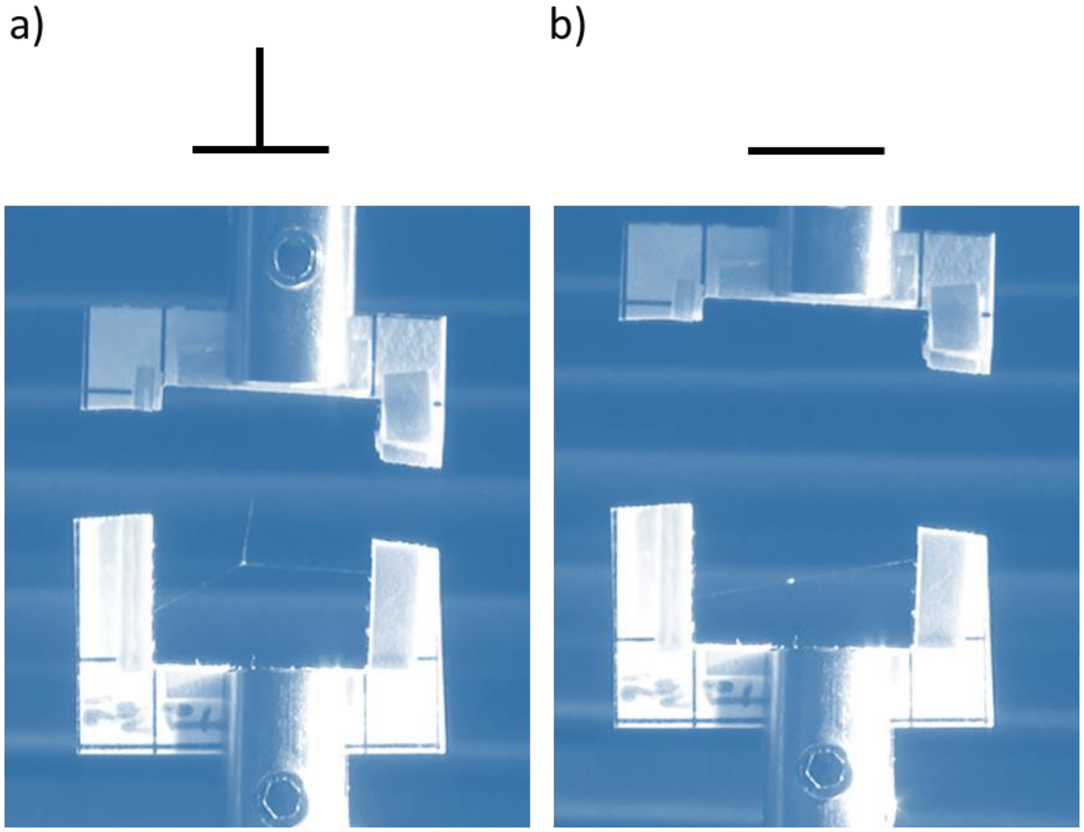

Figure S5: A TRR sample before (a) and after (b) the test.

Sample (TSR)	Force at break (mN)	Displacement at break (mm)	Energy dissipated at break (µJ)
1	0.74	8.99	3.26
2	0.13	10.26	0.15
3	0.56	4.15	1.20
4	0.43	11.26	1.00
5	0.33	14.90	1.86
6	0.51	11.19	1.12
7	0.42	5.37	1.90
8	0.37	7.11	1.03
9	0.18	7.00	0.36
10	0.31	13.75	3.14

Table S.1.: Results from tests on TSR sample.

Sample (CSR)	Force at break (mN)	Displacement at break (mm)	Energy dissipated at break (µJ)
1	0.91	10.26	2.00
2	1.13	5.85	2.93
3	1.13	6.20	6.42
4	0.92	5.74	3.03
5	0.90	17.36	2.95
6	0.71	14.13	2.24
7	0.66	11.97	5.80
8	0.62	8.05	1.52
9	0.98	15.40	4.29
10	1.01	2.97	1.12

Table S.2.: Results from tests on CSR sample.

Sample (NSR)	Force at break (mN)	Displacement at break (mm)	Energy dissipated at break (µJ)
1	1.72	15.15	4.02
2	1.59	20.00	10.29
3	0.65	21.02	16.62
4	1.26	22.71	7.10
5	1.11	10.86	3.98
6	0.85	24.75	5.19
7	1.66	20.97	4.16
8	0.73	18.56	13.74
9	1.65	14.27	4.04
10	1.36	24.78	4.45

Table S.3.: Results from tests on NSR sample.

Sample (TRR)	Force at break (mN)	Displacement at break (mm)	Energy dissipated at break (µJ)
1	7.35	5.12	14.26
2	7.70	4.85	7.16
3	12.87	3.22	29.78
4	7.55	5.55	21.65
5	7.70	4.91	19.06
6	10.00	5.00	12.57
7	9.85	3.44	25.17
8	10.16	5.00	28.56
9	9.44	4.98	17.65
10	11.80	6.59	12.40

Table S.4: Results from tests TRR sample.

Sample (RFS)	Force at break (mN)	Displacement at break (mm)	Energy dissipated at break (μJ)
1	10.01	1.90	36.19
2	24.70	1.56	10.96
3	15.57	2.32	33.20
4	31.15	3.04	34.73
5	38.13	4.20	43.45
6	15.05	3.03	25.89
7	14.40	1.78	16.29
8	9.30	2.26	14.43
9	15.96	3.33	24.03
10	9.91	5.17	26.25

Table S.5: Results from tests on RFS sample.

Compared samples	Ratio between dissipated energies
TRR/TSR	13
RFS/TRR	2.0
CSR/TSR	2.3
NSR/TSR	4.9
NSR/CSR	2.2

Table S.6: Comparison between the energy required to break different samples.

Compared samples	Ratio between detachment forces
TRR/TSR	23
RFS/TRR	2.0
CSR/TSR	2.4
NSR/TSR	3.3
NSR/CSR	1.4

Table S.7: Comparison between the detachment forces of different samples.

Compared samples	Ratio between displacement at break
TRR/TSR	0.56
RFS/TRR	0.58
CSR/TSR	1.9
NSR/TSR	2.1
NSR/CSR	1.4

Table S.8: Comparison between the displacements at break of different samples.

Statistical analysis

ANOVA Analysis

Analysis of variance was performed to compare the mean values of the forces at break. The parameters used to verify the null hypothesis, i.e. all the data sets come from the same distribution and have the same mean value, were

$$SSQ_a = \sum_{g=1}^{G} n_g \left(m_g - m\right)^2$$

$$SSQ_e = \sum_{g=1}^{G} \sum_{j=1}^{n_g} \left(x_{gj} - m_g\right)^2$$

Where *G* is the number of different samples under consideration, n_g is the number of tests of the same sample, *m* is the mean value of all the data, m_g is the mean value within the group (i.e., sample), and *x* is the single force value. These sums of squares were used to compute the T value

$$T = \frac{\frac{SSQ_a}{G-1}}{\frac{SSQ_e}{n-G}}$$

that has been compared with the ideal value of the Fisher function *F* with a significance level of 5%. If *T>F* we reject the null hypothesis and thus we can consider the difference among the data set as significant (i.e., the difference is due to intrinsic differences among the samples and not a consequence of internal variance). In our case, *g=10*, *G=5 or 2* (according to the number of samples considered in the comparison), *n=50 or 20* (according to the number of samples considered in the comparison). The p-value was computed with the support of MatLab®.

Table S9 shows that the difference among all the studied samples is significant. The relatively small difference in T value between CSR and NSR samples confirms the hypothesis that the magnitude of the force at break saturates with the increase in complexity (and thus stiffness) of the sample structure.

	ALL	TSR CSR	TSR NSR	TSR TRR	TSR RFS	CSR NSR	CSR TRR	CSR RFS	NSR TRR	NSR RFS	TRR RFS
SSQa	2459.30	5.72	3.70	408.97	1623.60	0.65	365.09	1534.93	1807.22	1472.33	402.84
SSQe	892.39	0.59	1.78	32.62	858.28	1.78	32.63	858.29	33.82	859.48	890.32
T	31.00	175.49	37.48	225.67	34.05	6.58	201.42	32.19	961.96	30.83	8.14
F	2.61	4.41	4.41	4.41	4.41	4.41	4.41	4.41	4.41	4.41	4.41
p-value	~0	~0	0.00000952	~0	0.00001596	0.01947250	~0	0.00002293	~0	0.00003345	0.01056178

Table S9: Values of the ANOVA analysis performed on the load at break of the different types of tested junctions. The first column reports the comparison among all the types of junction samples, while and the following columns report the comparison between two different samples (all combinations).

Weibull Statistics

Weibull statistics was also considered in order to evaluate the force at break distribution across our different samples. The main Weibull parameters, namely scale and shape parameters, were derived from a linear regression fit implemented in the Matlab® software environment.

We considered the cumulative density function, *CF*, defined as

$$CF(F, m, F_0) = 1 - e^{-\left(\frac{F}{F_0}\right)^m}$$

where *F* is the force at break of the sample, *m* is the shape parameter and F_0 the scale parameter.

For the linear regression fit, we used the median rank estimator

$$\widehat{CF}(F, M, F_0) = \frac{i - 0.3}{n + 0.4}$$

where *i* is the position of the sample with respect to the maximum among the data and *n* is the number of tested samples. From the double logarithm

$$\ln\left(ln\left(\frac{1}{1 - \widehat{CF}}\right)\right) = mln(F) - mln(F_0)$$

we can estimate the Weibull parameters *m* and F_0 (Figures S6-S10), which then allows to model the probability density distribution corresponding to the force at break of all our samples (Figure S11).

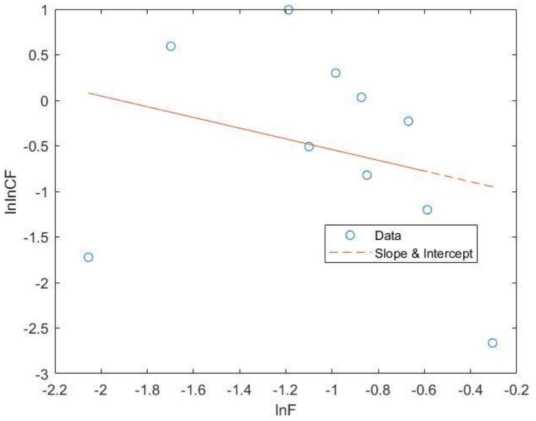

Fig S6: Weibull linear regression method applied to the TSR sample.

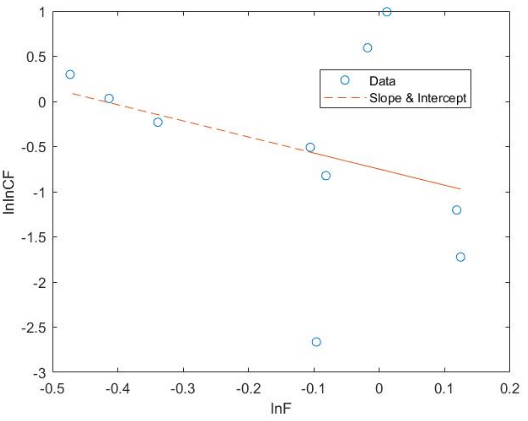

Fig S7: Weibull linear regression method applied to the CSR sample.

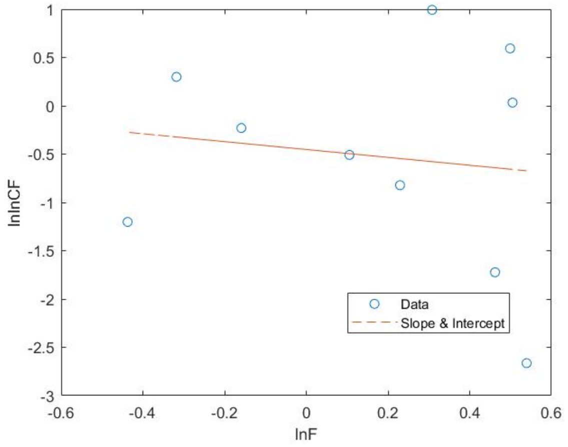

Fig S8: Weibull linear regression method applied to the NSR sample.

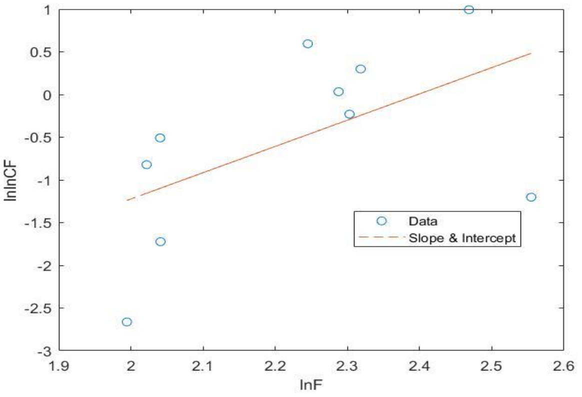

Fig S9: Weibull linear regression method applied to the TRR sample.

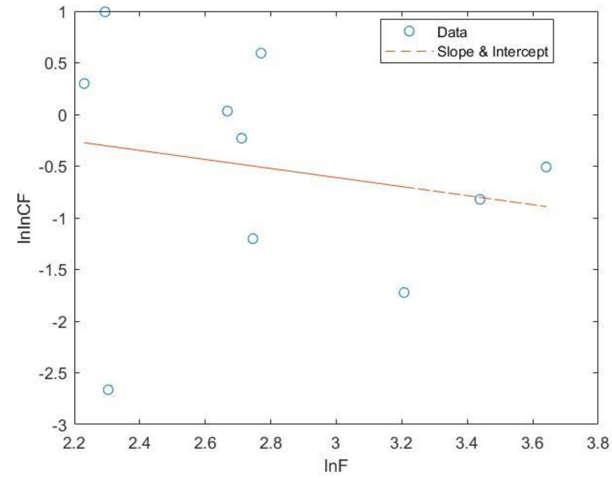

Fig S10: Weibull linear regression method applied to the RFS sample.

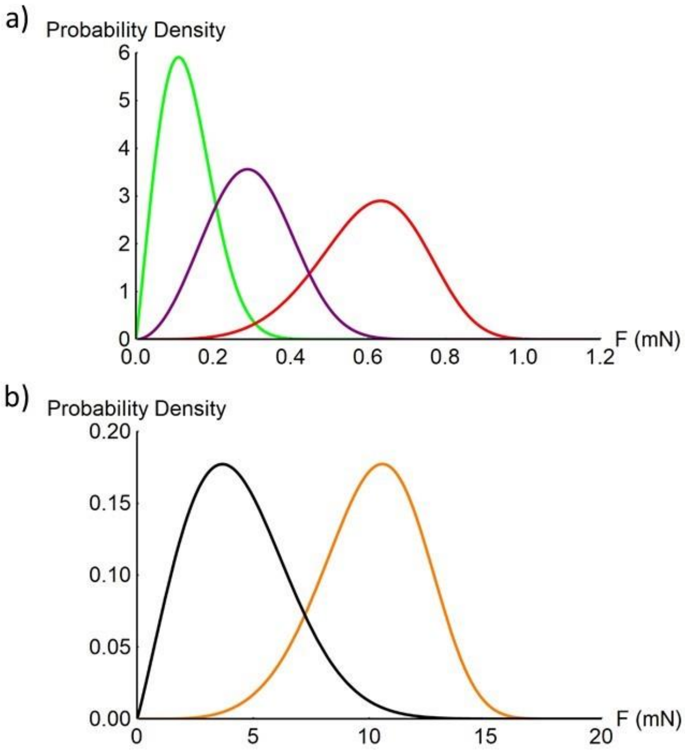

Fig S11: Weibull probability density function of the force required to break different web samples during a tensile test. a) TRS, CSR and NSR samples fail at forces significantly smaller than TRR and RFS samples (b). Note that in the first set of samples (a) a spiral thread is pulled out of the corresponding web sample during the test, while in the second set of samples (b) a radial thread is pulled out.
